# Moderate Normobaric Hypoxia Does Not Exacerbate Left Ventricular Dysfunction After Exhaustive Exercise in Athletes and Untrained Individuals

**DOI:** 10.3390/jcm14238391

**Published:** 2025-11-26

**Authors:** Robert Gajda, Kamila Płoszczyca, Ewa Kowalik, Adam Niemaszyk, Michał Starczewski, Natalia Grzebisz-Zatońska, Katarzyna Kaczmarczyk, Józef Langfort, Miłosz Czuba

**Affiliations:** 1 Department of Kinesiology and Health Prevention, Jan Dlugosz University, 42-200 Czestochowa, Poland; gajda@gajdamed.pl; 2 Center for Sports Cardiology, Gajda-Med Medical Center in Pultusk, 06-100 Pultusk, Poland; 3 Faculty of Rehabilitation, Józef Piłsudski University of Physical Education in Warsaw, 00-968 Warsaw, Poland; kamila.ploszczyca@awf.edu.pl (K.P.); adam.niemaszyk@awf.edu.pl (A.N.); michal.starczewski@awf.edu.pl (S.M.); natalia.grzebisz@awf.edu.pl (N.G.-Z.); katarzyna.kaczmarczyk@awf.edu.pl (K.K.); 4 Department of Congenital Heart Diseases, National Institute of Cardiology, 04-628 Warsaw, Poland; 5 Department of Sports Theory, Jerzy Kukuczka Academy of Physical Education, 40-065 Katowice, Poland; j.langfort@awf.katowice.pl

**Keywords:** exercise-induced cardiac fatigue, hypoxia, echocardiography, left ventricular function, athletes, endurance exercise

## Abstract

**Background:** Exposure to hypoxia is widely used to enhance training adaptations, but its acute effects on cardiac function remain unclear. Exercise-induced cardiac fatigue (EICF), defined as transient impairments in left ventricular (LV) systolic and diastolic function, has been reported after endurance exercise. Whether moderate hypoxia influences EICF, particularly in athletes, is unknown. **Methods:** Twenty-four healthy men participated: 12 endurance-trained cyclists (T) and 12 untrained individuals (UT). Each completed two exhaustive cycling tests under normoxia (FiO_2_ = 20.9%) and moderate normobaric hypoxia (FiO_2_ = 14.4%; ~3000 m). Echocardiography was performed at rest and immediately post-exercise to assess LV systolic and diastolic function. **Results:** Exhaustive exercise reduced LV diastolic function in both groups, with no significant condition-related differences. Under normoxia, early peak filling velocity (Mitral E) decreased by 21.2% in UT and 23.2% in T, and under hypoxia, by 16.2% in UT and 14.9% in T. Global longitudinal strain (LV GLS) became less negative after exercise under normoxia (UT: +25.2%, T: +30.6%) and hypoxia (UT: +24.8%, T: +20.3%). Athletes exhibited slightly less post-exercise systolic impairment under hypoxia than normoxia, reflected by the maintenance of a more negative LV GLS (∆GLS: 6.87 ± 2.65% in normoxia vs. 4.55 ± 1.86% in hypoxia, *p* < 0.05). **Conclusions:** Moderate normobaric hypoxia (~3000 m) did not exacerbate EICF in either group. Athletes showed slightly less post-exercise systolic impairment under hypoxia. Moderate hypoxia may modify the cardiac response to exhaustive exercise, but studies with larger samples and direct preload assessment are needed.

## 1. Introduction

In both exercise adaptation research and sports practice, training protocols that include exposure to hypoxic conditions are widely used. Such protocols can be applied either in a hypobaric environment (resulting from reduced barometric pressure) or in a normobaric environment (achieved by lowering the fraction of inspired oxygen). These interventions aim to improve physical performance under normoxic conditions and to accelerate acclimatization prior to exercise performed at high altitude [1,2,3]. In recent years, there has also been growing interest in the therapeutic applications of normobaric hypoxia, including its potential role in the prevention and treatment of metabolic and cardiovascular diseases [4,5,6].

Hypoxia is defined as a state in which oxygen delivery to the body is insufficient to meet current metabolic demands, resulting in impaired exercise capacity [7,8]. The key factor underlying reduced exercise tolerance under hypoxic conditions is a decrease in maximal oxygen uptake (VO_2max_). The decline in VO_2max_ is linked to a reduction in arterial oxygen partial pressure (PaO_2_), which limits oxygen transport to tissues, impairs metabolic processes, and reduces contractile function of both skeletal muscle and the myocardium [9,10]. Acute hypoxic exposure constitutes a major stressor for the cardiovascular system, triggering a range of physiological responses designed to preserve systemic oxygen delivery. At rest, sympathetic activation and increased catecholamine release lead to elevated heart rate (HR), higher blood pressure, and increased cardiac output (CO), typically without significant changes in stroke volume [11,12,13]. During maximal exercise under hypoxia, however, both maximal CO and maximal HR are reduced [14].

Exposure to hypoxia induces hypoxic pulmonary vasoconstriction and increases pulmonary artery pressure (PAP), which in turn elevates the afterload of the right ventricle (RV) [15]. These changes may lead to a slight impairment of left ventricular (LV) filling due to the mechanical constraint imposed by RV overload [13]. Previous studies in healthy humans have demonstrated that acute passive exposure to hypoxia is associated with improved LV systolic function and a mild reduction in diastolic function [16,17]. Animal studies further suggest that LV functional changes may depend on both the duration and severity of hypoxic exposure [18,19].

Another factor influencing LV functional and morphological changes is physical exercise [20,21]. Intense endurance exercise under normoxic conditions places a hemodynamic load on the RV and elevates PAP due to the mismatch between increased stroke volume and insufficient pulmonary vascular dilation. This results in reduced venous return and may ultimately impair LV diastolic function [22]. During prolonged endurance exercise, sustained high cardiac loading can lead to transient cardiac dysfunction, commonly referred to as exercise-induced cardiac fatigue (EICF) [23]. EICF manifests as a transient reduction in both LV systolic performance and diastolic filling [20,24]. Potential mechanisms underlying EICF during endurance exercise include myocardial cell damage, β-adrenergic down-regulation, elevated levels of free fatty acids leading to reduced inotropic state, increased production of reactive oxygen species, and altered Ca^2+^ metabolism [23].

High-intensity training sessions conducted under moderate hypoxia (FiO_2_ = 16.5–14.4%, ~2000–3000 m above sea level) are now a standard practice in many sports disciplines [25,26,27]. Moderate hypoxia provides an optimal balance between a sufficiently strong adaptive stimulus and its good tolerance, allowing for the maintenance of training quality [28,29,30]. Athletes performing intense and/or prolonged endurance training under hypoxic conditions are exposed to the combined effects of both hypoxia and exercise-induced cardiac load.

So far, the short-term consequences of performing an acute bout of strenuous exercise under hypoxic conditions remain poorly understood. In a recent study [31], we observed no differences in cardiac marker activity, including troponin I and T, heart-type fatty acid-binding protein (H-FABP), creatine kinase-MB isoenzyme (CK-MB), and myoglobin (Mb), after high-intensity interval exercise under normobaric hypoxia (FiO_2_ = 15.5%) compared to normoxia. Kleinnibbelink et al. [32] demonstrated that hypoxia (FiO_2_ = 14.5%, 3000 m) had no effect on the magnitude of exercise-induced cardiac fatigue in the left ventricle (LV) after 45 min of high-intensity exercise in healthy individuals. Kullmer et al. [33] showed that hypoxia (FiO_2_ = 14.0%) did not affect the morphology and function of the LV either at rest or during exercise. In contrast, research by Goebel et al. [34] revealed that during exercise under severe hypoxia (FiO_2_ = 9.9%; 5500 m), there was a reduction in LV contractile reserve compared to normoxic conditions. To the best of our knowledge, the available literature examining the effects of exercise on the LV focuses on untrained individuals. No study has yet investigated whether the LV’s response to intense exercise in hypoxic conditions differs between endurance athletes and untrained subjects. Therefore, the aim of this study was to investigate the acute effects of high-intensity exercise under moderate hypoxia (FiO_2_ = 14.4%) versus normoxia on left-sided cardiac function and morphology in healthy untrained individuals and endurance athletes.

## 2. Materials and Methods

### 2.1. Study Participants

The study involved 24 healthy male volunteers who were divided into two groups: 12 untrained individuals (UT group; age: 31.7 ± 8.3 years; height: 181.1 ± 6.5 cm; body mass: 85.7 ± 11.5 kg; body fat: 17.3 ± 6.3%; VO_2max_: 44.1 ± 7.4 mL∙kg^−1^∙min^−1^) and 12 trained male cyclist athletes (T group; age: 26.5 ± 7.7 years; height: 180.8 ± 3.6 cm; body mass: 71.4 ± 5.3 kg; body fat: 12.3 ± 2.6%; VO_2max_: 64.2 ± 2.9 mL∙kg^−1^∙min^−1^). Participants in T group met the following criteria: VO_2max_ not less than 60 mL/kg/min, training experience of at least 6 years and at least a six-month waiting period from any previous altitude training. Additionally, inclusion criteria for both groups were: (1) age between 20 and 40 years; (2) absence of chronic diseases; (3) systolic blood pressure between 100 and 140 mmHg and diastolic blood pressure between 60 and 90 mmHg. Exclusion criteria were (1) use of drugs, alcohol consumption, or smoking; (2) hypertension; (3) premature termination of the exercise test. An a priori power analysis was performed using G*Power 3.1. The calculation was based on our primary outcomes, global longitudinal strain (GLS) and LV EF. The expected effect size (partial *η*^2^) of 0.30 was derived from previous literature reporting large to very large effects in response GLS and LV EF to exercise [32]. Assuming a repeated-measures ANOVA design, α = 0.05 and power (1−β) = 0.80, the required sample size was n = 20. To account for an anticipated dropout rate of ~20%, we aimed to recruit n = 24 participants.

**Figure 1:**
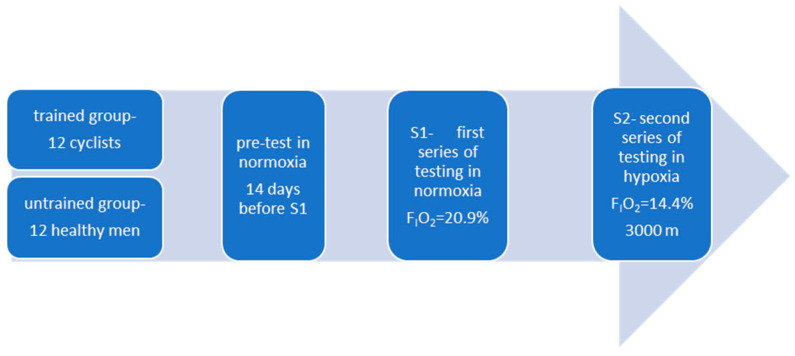
Illustration of the study design.

Prior to the main experiment, all participants performed an incremental test on a cycle ergometer under normoxic conditions (Figure 1).

Forty-eight hours before the test, the participants did not perform any physical exercise. During each testing series, the subjects were accommodated under controlled conditions for 48 h, and during this period their mixed diet (3000 kcal) and hydration—both before and after exercise—were regulated. During exercise, the athletes consumed water ad libitum. Before the test, hematocrit values were assessed using OPTI CCA-TS2 Blood Gas and Electrolyte Analyzer (OPTI Medical Systems, Roswell, GA, USA). No significant differences were found in hematocrit values (UT group: 45.93 ± 1.14% vs. 46.03 ± 1.08%; T group: 47.05 ± 2.1% vs. 47.2 ± 1.91%).

This preliminary test was used to determine VO_2max_ and verify that the basic criterion for participation in the T group (VO_2max_ not less than 60 mL/kg/min) was met, as well as to familiarize participants with the experimental protocol.

All subjects presented valid medical certificates confirming their good health and fitness for intensive physical exercise. They were fully informed about the study’s purpose and provided written consent to participate. The study was conducted in accordance with the principles of the Declaration of Helsinki and approved by the Bioethics Committee at the University of Zielona Góra, Poland (Resolution No. 21/2022, issued on 9 November 2022).

### 2.2. Study Design

The experiment consisted of two test series conducted under different environmental conditions: S1 (normoxia) and S2 (normobaric hypoxia) (Figure 1). The fraction of inspired oxygen was set at F_I_O_2_ = 20.9% for S1 (normoxia) and F_I_O_2_ = 14.4% for S2 (~3000 m a.s.l.). The chosen level of hypoxia was based on scientific evidence indicating that the 2000–3000 m range represents the “gold standard” for endurance training under hypoxic conditions. Moderate hypoxia is most frequently used in endurance training because higher altitudes carry an increased risk of overtraining and impair the athlete’s ability to respond effectively to both hypoxic and training stimuli [28,29,30].

The order of test series was randomized for each participant using an Excel random number generator. All trials were conducted in a normobaric hypoxic chamber (AirZone, AirSport, Poland). Before the start of the project and taking into account the baseline altitude of the study location (110 m above sea level), all sensors of the hypoxic system were calibrated. The system includes two independent oxygen sensors monitoring room O_2_ concentration, and it automatically shuts down if a discrepancy greater than 0.4% (~250 m of altitude difference) is detected. After reaching the target simulated altitude (FiO_2_), the hypoxic system continuously adjusted the gas mixture to maintain stable O_2_ and CO_2_ levels throughout testing. Environmental stability was further ensured by the large size of the hypoxic room (75 m^2^), individual exercise testing, and continuous monitoring of room conditions. During all test series, the atmospheric conditions in the hypoxic room were kept constant in regard to temperature (19 ± 0.5 °C), humidity (45–50%), concentration of carbon dioxide (700 ± 50 ppm), as well as concentration of oxygen (FiO_2_ = 14.4 ± 0.2%).

To maintain blinding, participants were not informed of the actual conditions (normoxia or hypoxia) in the chamber. Each series was carried out on a single day and consisted of two consecutive ergocycle tests, separated by a 10-min active recovery period (see Testing Protocol, below; Figure 1). The methodology was identical across both series, and all testing sessions were performed at the same time of day to minimize circadian variability. A one-week active recovery period separated the two series, during which participants avoided hypoxic exposure and refrained from high-intensity exercise.

To confirm full recovery before each session, baseline resting values for EF, GLS, and HR were recorded. The mean pre-session values were as follows: UT group (S1 vs. S2): LV EF = 65.71 ± 5.58% vs. 68.46 ± 5.16%, LV GLS = −19.95 ± 2.44% vs. −20.30 ± 2.45%, HR at rest = 72.9 ± 7.5 bpm vs. 74.2 ± 5.7 bpm, VO_2_ at rest = 564.1 ± 87.3 mL/min vs. 606.6 ± 90.8 mL/min; T group: LV EF = 67.1 ± 4.45% vs. 68.21 ± 4.83%, LV GLS = −22.41 ± 3.24% vs. −22.37 ± 2.34%, HR at rest = 63.7 ± 7.2 bpm vs. 66.0 ± 8.8 bpm, VO_2_ at rest = 595.8 ± 101.3 mL/min vs. 601.6 ± 104.2 mL/min (HR and VO_2_ at rest values were obtained from baseline ergospirometry measurements), with no significant differences between sessions (*p* > 0.05), indicating comparable baseline conditions across visits.

### 2.3. Testing Protocol

In series S1, body mass and body composition were measured between 7:00 and 7:30 a.m., prior to breakfast. Body height was assessed using an anthropometer with an accuracy of 0.5 cm, and body composition was estimated using bioelectrical impedance analysis (InBody 220, Biospace, Singapore).

In both series (S1 and S2), participants consumed a light mixed meal (5 kcal∙kg^−1^ body weight; 50% carbohydrates, 30% fat, 20% protein) two hours before testing. Afterward, they underwent 20 min of passive exposure to the assigned condition (normoxia or hypoxia) before performing two consecutive ergocycle tests, separated by a 10-min active recovery period. All exercise was performed on a cycle ergometer (Excalibur Sport, Lode BV, Groningen, the Netherlands), adjusted individually for each participant.

The first test in each series was a graded exercise test (GXT) to volitional exhaustion. Exercise began at 90 W, with workload increments of 30 W every three minutes until exhaustion. At the end of each stage (last 15 s), capillary blood samples were collected from the fingertip to measure blood lactate concentration (LA; LABTREND, BST Bio Sensor Technology GmbH, Isernhagen, Germany). These data were used to analyze lactate kinetics and to determine the individual lactate threshold (WR_LT_) using the D_max_ method [35]. Following the GXT, participants completed a 10-min active recovery session at 30% of their maximum workload reached during exhaustion (WR_max_).

Subsequently, a constant-workload exercise test (CXT) was performed at WR_LT_, continued until volitional exhaustion. During the CXT, participants were allowed to drink water ad libitum. Capillary blood samples were collected before and immediately after the test to determine LA concentration and acid–base balance (OPTI CCA-TS2 Blood Gas and Electrolyte Analyzer, OPTI Medical Systems, Roswell, GA, USA).

Throughout all tests, heart rate (HR) and oxygen saturation (SpO_2_) were continuously monitored at rest and during both the GXT and CXT using a pulse oximeter (WristOx2, Nonin Medical Inc., Plymouth, MN, USA).

### 2.4. Transthoracic Echocardiography (TTE)

In both series (S1, S2), participants underwent two complete transthoracic echocardiographic (TTE) examinations: at rest after 20 min of passive exposure to the selected assigned condition (normoxia or hypoxia) and at the second minute after the two consecutive ergocycle tests. All echocardiographic assessments were performed using the EPIQ system (Philips Medical Systems, Andover, MA, USA) and an X5-1 phased-array transducer, with the participant in the left lateral decubitus position. Echocardiographic measurements were obtained by a cardiologist experienced in echocardiography (E.K.) following current guidelines [36,37]. All analyses were performed by the same researcher who was blinded to participant status. Echocardiographic image acquisition was carried out by an unblinded operator to ensure optimal technical accuracy under hypoxic conditions, with the operator returning to normoxia after each assessment to avoid inaccuracies related to prolonged hypoxic exposure; all imaging analyses were subsequently completed by a blinded researcher.

**Figure 2:**
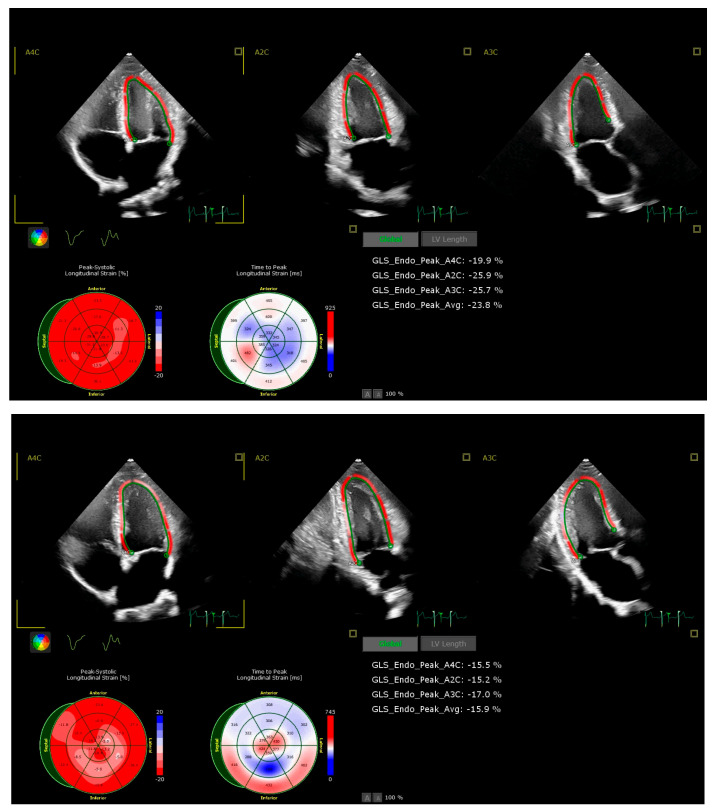
Examples of echocardiographic assessment of LV GLS in an untrained participant at rest (upper panel, GLS −23.8%) and post-exercise (lower panel, GLS −15.9%) in hypoxic conditions.

The left ventricular end-diastolic (LVEDV) and end-systolic (LVESV) volumes, as well as LV ejection fraction (LV EF), were assessed using the modified Simpson’s biplane method. To assess LV systolic function, peak LV myocardial systolic velocities at the mitral annulus level (S′) were also measured. Care was taken to acquire images with optimal technical setting parameters and a frame rate between 50–90 fps for speckle-tracking analysis. LV global longitudinal strain (GLS) was calculated using an 18-segment model from apical four-, three- and two-chamber views, using dedicated conventional software (QLab v.15.5, Philips Medical Systems) (Figure 2). For the assessment of diastolic function, early peak diastolic filling velocity (E) and late peak diastolic filling velocity (A) were obtained using the pulsed Doppler transmitral flow from the apical four-chamber view. Additionally, peak LV myocardial early (E′) and late (A′) diastolic velocities were recorded using tissue Doppler imaging at the mitral annulus level, with averages taken from the septal and lateral walls (E′ mean and A′ mean, respectively). Subsequently, the ratio of transmitral E velocity to early diastolic mitral annular velocity (E/E′) and the ratio of early to late annular velocity (E′/A′) were calculated to determine LV diastolic function. Left atrial (LA) diameter was measured at LV end-systole from the parasternal long-axis view. LA volume was measured using the biplane Simpson’s disk method from both the apical two- and four-chamber views at the end of ventricular systole and indexed by body surface area (LAVi).

Intra-observer reproducibility of LV myocardial deformation and pulsed Doppler measurements was evaluated in a subset of 10 randomly selected subjects. It was expressed as percentage of error, derived as the absolute difference between 2 sets of measurements divided by the mean of the observations and using intraclass correlation coefficients (ICC). Intra-observer measures were performed at least 2 months apart.

### 2.5. Statistical Analysis

All data were collected and analyzed using Statistica software v.13 (StatSoft, Hamburg, Germany). Statistical significance was set at *p* < 0.05 for all analyses. Prior to statistical testing, the Shapiro–Wilk test was applied to assess the normality of variable distributions, and Levene’s test was used to verify homogeneity of variance. Sphericity was assessed using Mauchly’s test. When the assumptions for parametric testing were satisfied, a two-way repeated-measures ANOVA was conducted. Effect sizes (partial *η*^2^) were calculated for all significant ANOVA results, and 95% confidence intervals (CIs) for the partial *η*^2^ were reported.

To examine specific pairwise comparisons (UT vs. T under the same condition; rest vs. post-exercise within each group under the same condition; normoxia vs. hypoxia within each group), contrast analysis was performed. The contrasts were conducted as planned comparisons using paired-sample *t*-tests (for within-subject factors) and independent-samples *t*-tests (for between-group factors) as appropriate. For primary outcomes (echocardiographic parameters), planned contrasts were grouped into three families (1) between-group differences at each environment × time combination (k = 4), (2) exercise effect (pre vs. post within each group × environment; k = 4), and (3) environment effect (normoxia vs. hypoxia within each group × time; k = 4). *p*-values were adjusted within each family using the Bonferroni correction (α_adj = 0.05/4 = 0.0125). Both raw and Bonferroni-adjusted *p*-values are reported.

## 3. Results

### 3.1. Changes in Mechanical Work, Heart Rate and Saturation of Hemoglobin During Exercise to Exhaustion Under Normoxia and Hypoxia

The mechanical work during both tests (GXT+CXT, W_mech_) was found to be significantly reduced (*p* < 0.01) in both groups under hypoxic conditions—by 31.5% in the UT group and 18% in the T group. W_mech_ was significantly higher (*p* < 0.001) in the T group compared to the UT group, again in both normoxia (36% higher) and hypoxia (43%). In both groups, hypoxia induced a significant decrease (*p* < 0.001) in heart rate recorded at the end of exercise performed to exhaustion (HR_end_), by approx. 4.5%. In addition, HR_end_ values in the T group were significantly higher (*p* < 0.05) than in the UT group in both conditions. However, when these values were converted to a percentage of maximum heart rate for the given conditions (%HR_max_con), no significant differences were observed between groups and conditions. Under hypoxia, there was also a significant reduction (*p* < 0.001) in hemoglobin saturation registered at the end of exercise (SpO_2end_) in both the UT (12%) and T (10%) groups. No significant changes in delta values of La (∆La) or pH (∆pH) in response to exercise to exhaustion were observed between conditions or groups (Table 1).

### 3.2. Echocardiography-Derived LV Dimensions and Systolic Function Parameters

Analysis of selected parameters of LV dimensions and systolic function showed no significant differences in resting values either between groups or conditions (normoxia vs. hypoxia).

Exercise to exhaustion in the UT group induced significant changes in interventricular septum (IVS) thickness (*F* = 25.656; *p* = 0.001; partial *η*^2^ = 0.54 (95% CI: 0.31–0.70)) and LV end-diastolic diameter (LVEDD; *F* = 11.702; *p* = 0.002; partial *η*^2^ = 0.35 (95% CI: 0.16–0.53)). In the T group, exercise to exhaustion resulted in significant changes in LVESD (*F* = 8.972; *p* = 0.007; partial *η*^2^ = 0.29 (95% CI: 0.12–0.48)), LV ejection fraction (LV EF; *F* = 16.190; *p* = 0.001; partial *η*^2^ = 0.42 (95% CI: 0.23–0.59)), septal S′ (*F* = 26.057; *p* = 0.001; partial *η*^2^ = 0.54 (95% CI: 0.33–0.71)), and lateral S′ (*F =* 8.354; *p* = 0.001; partial *η*^2^ = 0.275 (95% CI: 0.11–0.46)). In both groups, exercise produced significant alterations in LVEDV (UT: *F* = 14.328, *p* = 0.001, partial *η*^2^ = 0.394 (95% CI: 0.20–0.57); T: *F* = 7.183, *p* = 0.013, partial *η*^2^ = 0.246 (95% CI: 0.08–0.43)) and LV GLS (UT: *F* = 88.433, *p* = 0.001, partial *η*^2^ = 0.801 (95% CI: 0.68–0.89); T: *F* = 149.664, *p* = 0.001, partial *η*^2^ = 0.872 (95% CI: 0.78–0.94)). Moreover, in the T group, hypoxic conditions significantly influenced LV GLS responses (*F* = 6.163; *p* = 0.021; partial *η*^2^ = 0.219 (95% CI: 0.06–0.40)).

Further analysis showed a significant increase in IVS thickness after exercise in the UT group under both normoxia (+4.6%; *t* = 4.62, *p* < 0.001, *p_Bonf* = 0.003). Under hypoxia, the UT group also demonstrated a significant reduction in LVEDD (−3.2%; *t* = 5.33, *p* < 0.001, *p_Bonf* = 0.001) and LVEDV (−9.3%; *t* = 6.04, *p* < 0.001, *p_Bonf* = 0.0003), whereas these changes were absent in the T group. The T group exhibited significant reductions in LV EF under normoxia (−7.1%; *t* = 3.83, *p* < 0.01, *p_Bonf* = 0.011) and septal S′ under hypoxia (−12.4%; *t* = 5.68, *p* < 0.001, *p_Bonf* = 0.0006). Lateral S′ decreased significantly only under normoxia (−15.9%; *t* = 3.14, *p* < 0.01, *p_Bonf* = 0.038) in the T group (Table 2).

**Figure 3:**
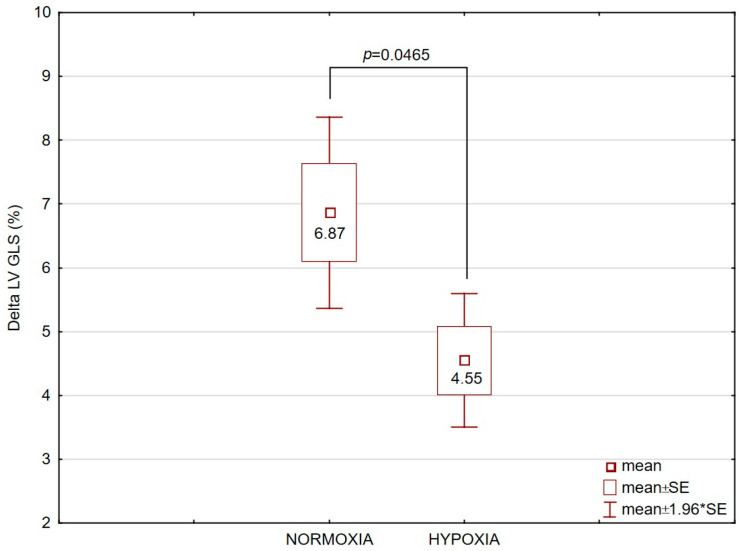
Delta (∆) values of left ventricular global longitudinal strain (LV GLS), expressed in percentage points (pp), after exercise to exhaustion in the trained (T, n = 12) group under normoxic and hypoxic conditions. Data are presented as mean ± SE. The comparison between conditions yielded *p* = 0.0465.

Finally, LV GLS increased significantly (*p* < 0.01) after exercise to exhaustion in both groups under normoxia (UT: +25.2%, *t* = 6.67, *p* < 0.001, *p_Bonf* = 0.0001; T: +30.6%, *t* = 8.99, *p* < 0.001, *p_Bonf* < 0.0001) and hypoxia (UT: +24.8%, *t* = 6.62, *p* < 0.001, *p_Bonf* = 0.001; T: +20.3%, *t* = 8.49, *p* < 0.001, *p_Bonf* < 0.0001). However, in the T group, the ∆LV GLS increase after exercise was significantly smaller under hypoxia compared with normoxia (Figure 3). No such differences were observed in the UT group.

### 3.3. Echocardiographic Parameters of LV Diastolic Function and LA Dimensions

Analysis of selected parameters of LV diastolic function and LA dimensions showed no significant changes in resting values either between groups or conditions (normoxia vs. hypoxia).

Exercise to exhaustion in T group resulted in significant changes in early ventricular filling velocity (Mitral E, *F* = 38.693, *p* = 0.001, partial *η*^2^ = 0.64 (95% CI: 0.348–0.773), E/A ratio (E/A, *F* = 12.811, *p* = 0.001, partial *η*^2^ = 0.37 (95% CI: 0.071–0.592), septal early diastolic mitral annulus velocity (E′septal, *F* = 30.743, *p* = 0.001, partial *η*^2^ = 0.58 (95% CI: 0.276–0.738), lateral early diastolic mitral annulus velocity (E′lateral, *F* = 22.693, *p* = 0.001, partial *η*^2^ = 0.51 (95% CI: 0.190–0.689), mean early diastolic mitral annulus velocity (Averaged E′, *F* = 33.618, *p* = 0.001, partial *η*^2^ = 0.60 (95% CI: 0.303–0.752), left atrial diameter (LAdiam, *F* = 8.689, *p* = 0.007, partial *η*^2^ = 0.28 (95% CI: 0.025–0.527), left atrial area (LA area, *F* = 11.571, *p* = 0.002, partial *η*^2^ = 0.34 (95% CI: 0.056–0.574) and left atrial indexed volume (LAVi, *F* = 27.215, *p* = 0.001, partial *η*^2^ = 0.55 (95% CI: 0.240–0.719) (Table 3). In the UT group, statistically significant changes were demonstrated in response to exercise for the following variables: Mitral E (*F* = 27.093, *p* = 0.001, partial *η*^2^ = 0.55 (95% CI: 0.238–0.718), E/A (*F* = 7.305, *p* = 0.013, partial *η*^2^ = 0.24 (95% CI: 0.012–0.500), E′septal (*F* = 12.464, *p* = 0.001, partial *η*^2^ = 0.46 (95% CI: 0.067–0.587), E′lateral (*F* = 14.207, *p* = 0.001, partial *η*^2^ = 0.39 (95% CI: 0.088–0.609), Averaged E′ (*F* = 19.752, *p* = 0.001, partial *η*^2^ = 0.47 (95% CI: 0.155–0.665), LAdiam (*F* = 9.377, *p* = 0.005, partial *η*^2^ = 0.30 (95% CI: 0.032–0.539), LA area (*F* = 17.453, *p* = 0.001, partial *η*^2^ = 0.44 (95% CI: 0.127–0.644) and LAVi (*F* = 30.883, *p* = 0.001, partial *η*^2^ = 0.58 (95% CI: 0.277–0.739).

Further analysis showed a significant decrease in Mitral E after exercise under both normoxia and hypoxia. Under normoxia, Mitral E decreased by 21.2% in the UT group (*t* = 3.75, *p* < 0.01, *p_Bonf* = 0.013) and by 23.2% in the T group (*t* = 4.52, *p* < 0.001, *p_Bonf* = 0.004). Under hypoxia, Mitral E also decreased significantly by 16.2% in UT (*t* = 3.59, *p* < 0.01, *p_Bonf* = 0.017) and by 14.9% in T (*t* = 4.43, *p* < 0.01, *p_Bonf* = 0.004).

Significant reductions in the E/A ratio (*p* < 0.05) were found only under normoxic conditions, with decreases of 24.7% in T (*t* = 3.49, *p* < 0.01, *p_Bonf* = 0.021).

A significant decreases in early diastolic mitral annulus velocity were noted after exercise to exhaustion in T group under normoxia (E′septal: −21.0%; (*t* = 4.70, *p* < 0.001, *p_Bonf* = 0.003), E′lateral: −19.1%; (*t* = 4.03, *p* < 0.01, *p_Bonf* = 0.008), averaged E′: −19.9%; *t* = 5.01, *p* < 0.001, *p_Bonf* = 0.002) and under hypoxia (E′septal: −13.9%; *t* = 3.09, *p* < 0.05, *p_Bonf* = 0.04), averaged E′: −12.5%; *t* = 3.16, *p* < 0.01, *p_Bonf* = 0.037). In the UT group, exercise caused a significant decrease in averaged E′ in normoxia (−17.4%; *t* = 3.44, *p* < 0.01, *p_Bonf* = 0.022). Although the magnitude of reduction in E′septal, E′lateral, and averaged E′ appeared smaller in the T group under hypoxia compared with normoxia, statistical analysis revealed no significant differences in the ∆ values between conditions (normoxia vs. hypoxia).

Interestingly, a significant decrease in LA diameter after exercise to exhaustion was observed only in the T group under normoxia (−6.1%; *t* = 3.27, *p* < 0.01, *p_Bonf* = 0.030). In contrast, in the UT group, a significant decrease in LA diameter was found under hypoxia (−4.8%; *t* = 3.08, *p* < 0.05, *p_Bonf* = 0.042), whereas this change was not present in the T group. In the T group, conditions significantly influenced LA diameter responses (*F* = 6.759, *p* = 0.016, partial *η*^2^ = 0.24 (95% CI: 0.010−0.485). Specifically, the post-exercise ∆LA diameter was significantly smaller under hypoxia compared with normoxia (Table 4). Such differences were not observed in the UT group. LA area decreased significantly after exercise in UT group under normoxia (−11.9%; *t* = 3.15, *p* < 0.01, *p_Bonf* = 0.037). Likewise, LAVi decreased significantly under normoxia (UT: −21.3%, *t* = 4.21, *p* < 0.01, *p_Bonf* = 0.006; T: −14.9%, *t* = 6.07, *p* < 0.001, *p_Bonf* = 0.0003) and hypoxia (UT: −18.6%, *t* = 3.66, *p* < 0.01, *p_Bonf* = 0.015).

### 3.4. Post Hoc Power Analysis

For all significant ANOVA results we calculated effect sizes (partial *η*^2^) together with 95% confidence intervals (CIs) to quantify the magnitude and precision of the observed effects. Observed partial *η*^2^ values ranged from 0.219 to 0.872, while the 95% CIs across effects extended approximately from 0.012 to 0.935, indicating medium to extremely large effects; for a subset of effects the 95% CIs were contained within the range ≈0.012–0.50. Post hoc power analysis was conducted using G*Power 3.1, assuming N = 12 per group and α = 0.05. The analysis indicated that the observed effects had estimated power ranging from 0.82 to >0.99. These calculations indicate that the study was adequately powered only to detect large effects. The observed CIs—including values near zero for some outcomes—indicate insufficient power to detect subtle physiological differences; therefore, future studies with larger sample sizes are recommended.

### 3.5. Reproducibility

Intra-observer variability for the early ventricular filling velocity (mitral E) was 3.2% at rest (ICC = 0.9809; 95% CI = 0.918–0.9998) and 4.0% post exercise (ICC = 0.9567; 95% CI = 0.6401–0.9954). For LV global longitudinal strain (GLS), the intra-observer variability at rest and after exercise was 4.7% (ICC = 0.9023; 95% CI = 0.4496 to 0.9891) and 5.8% (ICC = 0.936; 95% CI = 0.6984–0.9991), respectively.

## 4. Discussion

### 4.1. Passive Exposure to Hypoxia

Short-term passive exposure to moderate hypoxia (20 min) did not significantly affect LV function, LV morphology, or LA dimensions. Functional and morphological changes in the LV under passive hypoxia appear to depend on both the duration and severity of exposure. Animal studies have shown that severe hypoxia (FiO_2_ = 10%) induces depression of LV function. Specifically, reductions in LV systolic pressure and LV contractility were observed only after 6 h of exposure, whereas after 1.5 h these parameters remained at normoxic levels [19]. In another study, short-term (30 min) exposure to 10% hypoxia enhanced LV contractility as well as systolic and diastolic functions. A decline in LV peak systolic pressure was observed only when oxygen concentration was lowered to 6.3% and exposure was extended to 45 min [18].

In human studies, Huez et al. [16] reported that passive exposure to hypoxia (4500 m) in healthy individuals was associated with improved LV systolic function (increased LV ejection fraction, isovolumic contraction wave velocity, acceleration, and systolic ejection wave velocity at the mitral annulus), along with altered diastolic function. These alterations included reduced tricuspid and mitral inflow and a lower E/A ratio, resulting from an increased atrial contraction (A wave). Similarly, Boos et al. [17] observed that acute hypoxia (180 min, ~4800 m) led to an increase in mitral valve inflow A velocity and a corresponding reduction in the E/A ratio, though without changes in LV filling indices measured by tissue Doppler echocardiography or in estimated LV filling pressure (E/E′). The authors concluded that these changes were mild and did not indicate overt diastolic dysfunction. In our study, no resting changes in LV diastolic function were observed under hypoxia, most likely due to the very short exposure time [19,38] and the relatively mild hypoxic stimulus (~3000 m).

### 4.2. Exercise Under Normoxic and Hypoxic Conditions

#### 4.2.1. LV Systolic Function and LV Dimensions

With regard to systolic function, both groups demonstrated an increase in LV global strain (LV GLS becoming less negative) following exercise under both normoxia and hypoxia. In addition, the T group showed a reduction in LV ejection fraction (LV EF) and a decrease in peak systolic mitral annulus velocity. These findings are consistent with those of Kleinnibbelink et al. [32], who reported reductions in LV systolic function (↓LV EF, less negative LV GLS) after high-intensity exercise under both normoxia and hypoxia (3000 m). They are also in line with other studies describing alterations in LV function after relatively short bouts of high-intensity exercise in normoxic conditions [21,39,40].

Exercise also induced changes in LV dimensions, which varied according to environmental conditions and participants’ training status. In the UT group, post-exercise LVEDV decreased under hypoxia. Similar decreases in LVEDV have previously been reported in healthy individuals following strenuous running exercise performed under both normoxia and hypoxia [32]. Our results also align with other studies showing that exercise reduces LVEDV [41,42,43,44,45]. This phenomenon has been attributed to the shortening of diastolic filling time, delayed myocardial relaxation due to heightened sympathetic activity, and increased pressure load during maximal exercise [46]. The variability of 2D-derived LVEDV measurements may reach 5–10%. The observed change in untrained individuals falls within this range, suggesting that the finding may reflect normal measurement variability rather than a true physiological response. Therefore, this result should be interpreted with caution.

#### 4.2.2. LV Diastolic Function and LA Dimensions

Our results revealed that intense physical exercise led to a decline in LV diastolic function, while moderate hypoxia did not further exacerbate this diastolic impairment. Numerous studies have reported a reduction in LV diastolic performance following exercise under normoxic conditions [20,47,48,49]. These changes are transient, with LV diastolic indices returning to baseline within a 24-h recovery period [24].

In our study, the post-exercise reduction in LA-to-LV blood flow during early diastole (decrease in Mitral E) and the impairment of LV relaxation (decrease in E′septal, E′lateral, and averaged E′) did not differ between normoxia and hypoxia.

Our results differ from those of Goebel et al. [34], who reported a post-exercise increase in Mitral E and E′septal under normoxia, with no changes observed under hypoxic conditions. The discrepancy may be attributed to the differing levels of hypoxic stimuli applied (3000 m vs. 4000–5500 m). Several other studies have reported the impact of severe hypoxia (>4500 m) on impairment of LV diastolic function [16,17]. However, there is limited data on the effects of moderate hypoxia on LV function, and the results from these studies are inconsistent. Kullmer et al. [33] demonstrated a reduction in LV diastolic function by approximately 8%, both at rest and during exercise under moderate hypoxia (FiO_2_ = 14%), compared to normoxia. In contrast, Kleinnibbelink et al. [32] found no effect of hypoxia (FiO_2_ = 14.5%) on the exercise-induced reduction in LV function and mechanics following 45 min of high-intensity running. On the other hand, Yan et al. [50] showed that acute hypoxia improved LV diastolic function during moderate-intensity cycling.

It should be noted that, in addition to the severity of the hypoxic stimulus, exercise intensity may play an important role in the LV response to hypoxia. In the study by Kullmer et al. [33], participants performed exercise of moderate intensity (incremental non-exhaustive cycle ergometry, 6-min increments of 50 W, maximal load 150 W), whereas in our study and in that of Kleinnibbelink et al. [32], exercise was carried out at high intensity (cycling to exhaustion at lactate threshold; 45 min of running at 85% HRmax). It is possible that high-intensity exercise under normoxia induces changes in LV diastolic function [20,23,47] that are not further exacerbated by a hypoxic stimulus [32]. This may be particularly relevant given that hypoxia enhances local fatigue [51,52], potentially leading to premature termination of exercise before the heart reaches its maximal workload. This assumption, however, requires verification in future studies directly comparing hypoxic exercise protocols of varying intensity.

In our study, we also demonstrated that intense exercise induced changes in LA dimensions in both the UT and T groups, which is consistent with previous findings [32]. Earlier studies by Furukawa et al. [53] reported that LA dimensions increase during moderate exercise but decrease at peak exercise, subsequently returning to baseline after recovery. One of the mechanisms contributing to reduced LA dimensions during intense exercise is its more efficient emptying, which represents a physiological response to the body’s increased oxygen demand during exertion. This effect is largely attributable to a substantial rise in HR and the associated shortening of diastolic filling time [54,55].

### 4.3. Trained vs. Untrained

Within-condition analysis (separately for normoxia and hypoxia, Table 4) showed that the cardiac response to intense exercise differed in some respects between untrained individuals and athletes. Athletes (T) exhibited a generally larger acute post-exercise systolic impairment (larger decrease in LV EF and Septal S′) compared with untrained individuals (UT). These findings suggest that the athlete’s heart may exhibit a higher susceptibility to short-term systolic fatigue when exposed to maximal exercise loads. It is noteworthy that in the T group, mechanical work during the exercise trials was significantly higher than in the UT group under both conditions. Previous reports on lower-intensity aerobic exercise suggested no differences in systolic or diastolic function between sedentary and trained individuals [23] or indicated that prolonged aerobic exercise (60–150 min) may serve as a sufficient stimulus to induce exercise-induced cardiac fatigue (EICF) in untrained but not in trained individuals [24]. The discrepancy likely stems from the use of high-intensity, exhaustive exercise in our study. We suspect that the athlete’s heart may be more susceptible to short-term systolic fatigue, potentially because it operates at substantially higher intensities during maximal exercise compared with untrained individuals. Athletes typically generate greater cardiac output, larger LV filling and emptying volumes, and higher volume and pressure loads [56,57,58,59], which may together increase the likelihood of transient cardiac “overload”.

A critical methodological consideration is that we did not perform a three-way Group × Exercise × Condition interaction analysis. Consequently, although within-condition comparisons indicate that athletes showed larger post-exercise declines in systolic function than untrained individuals, these differences cannot be interpreted as condition-dependent group effects. Instead, our findings should be understood as differences that appeared within each environmental condition.

Importantly, a key finding of our study is that moderate hypoxia (3000 m) did not exacerbate EICF. Moreover, athletes exhibited slightly less post-exercise systolic impairment under hypoxic conditions than under normoxic conditions, as reflected by the maintenance of a more negative LV GLS. A likely explanation for the reduced cardiac load during exhaustive exercise in hypoxia is the accentuated peripheral fatigue of locomotor muscles, which leads to earlier exercise termination before the heart reaches maximal loading, thereby protecting the myocardium from excessive exercise-induced functional decline. There are suggestions that intra-subject variability should be considered when interpreting small differences in GLS. Nevertheless, it is important to clarify that GLS is a highly sensitive marker of early systolic impairment in individuals with preserved global systolic function. Current recommendations (e.g., AHA Scientific Statement on Speckle-Tracking Strain Echocardiography) indicate that a relative change in GLS of approximately 15% from baseline is considered clinically meaningful. In our study, the observed between-condition differences in ∆GLS in athletes approached this threshold, suggesting that they may represent early beneficial changes rather than simple measurement variability. Our proposed explanation—e.g., reduced maximal cardiac load due to the earlier onset of peripheral fatigue in hypoxia—remains somewhat speculative, especially in the absence of concurrent hemodynamic or oxygen delivery data in our study.

### 4.4. Limitations

Our study has several limitations. First, the relatively small sample size limits the generalizability of our findings and limits sensitivity to small effects (*ηp*^2^ < ~0.10–0.15). As the study cohort consisted exclusively of healthy males aged 20–40 years, the results cannot be directly extrapolated to other populations, particularly individuals with asymptomatic or preclinical conditions. Second, the duration of hypoxic exposure was relatively short and may not fully reflect the physiological adaptations observed during prolonged altitude exposure. However, the applied research model corresponds to real-world training practice involving intermittent hypoxic training. Third, although exercise intensity was individually adjusted to ensure comparable metabolic strain in normoxia and hypoxia, the mechanical workload (power output) achieved in hypoxia was lower due to physiological limitations associated with reduced oxygen availability. This undoubtedly influenced the results and must be considered when interpreting the data.

Additionally, due to the absence of a complete tricuspid regurgitation Doppler jet in most participants, we were unable to reliably estimate RV systolic pressure and therefore could not assess right-ventricular hemodynamics or potential RV–LV interdependence. We also did not analyze LV radial or circumferential deformation, focusing exclusively on longitudinal deformation, as GLS is the most widely used and sensitive clinical marker of early systolic dysfunction. Finally, imaging analysis was performed by a single reader, and intra- and interobserver variability could not be assessed.

## 5. Conclusions

Moderate hypoxia did not exacerbate transient post-exercise changes in LV function in either athletes or untrained individuals. Athletes showed slightly less post-exercise systolic impairment under hypoxia. Moderate hypoxia may modify the cardiac response to exhaustive exercise, but studies with larger samples, direct preload assessment, and incorporate longitudinal monitoring are needed.

## Figures and Tables

**Table 1:** Selected physiological and metabolic variables during exercise to exhaustion under normoxia and hypoxia in the untrained (UT, n = 12) and trained (T, n = 12) group. All values shown as ±SD.

Variable	Group	Normoxia	Hypoxia (3000 m)	Change
W_mech_ (kJ)	UT	534.62 ± 173.96	434.94 ± 144.37 ***	−18.65%
	T	937.83 ± 183.14 ^###^	762.06 ± 165.57 **^###^	−18.74%
W_mech_ (kJ/kg)	UT	6.28 ± 1.92	5.12 ± 1.6 ***	−18.47%
	T	13.08 ± 2.06 ^###^	10.71 ± 2.41 **^###^	−18.12
HR_end_ (bpm)	UT	172.41 ± 8.71	164.41 ± 7.58 ***	−4.64%
	T	179.33 ± 7.41 ^#^	171.75 ± 8.51 ***^#^	−4.23%
%HR_max_con	UT	95.65 ± 2.72	96.07 ± 2.96	+0.44%
	T	95.35 ± 2.23	95.48 ± 2.57	+0.14%
SpO_2end_ (%)	UT	96.16 ± 1.11	84.91 ± 2.39 ***	−11.70%
	T	94.16 ± 1.11	84.41 ± 4.37 ***	−10.35%
∆La (mmol/L)	UT	6.51 ± 1.9	6.35 ± 2.35	−2.46%
	T	5.17 ± 1.72	4.75 ± 1.72	−8.12%
∆pH	UT	−0.114 ± 0.048	−0.101 ± 0.041	−11.40%
	T	−0.091 ± 0.026	−0.075 ± 0.042	−17.58%

Abbreviations: W_mech_—total mechanical work performed during both tests (GXT + CXT), HR_end_—heart rate recorded at the end of exercise performed to exhaustion, %HR_max_con—% of maximum heart rate under the given conditions, SpO_2end_—hemoglobin saturation registered at the end of exercise, ∆La—change in blood lactate concentration in response to exercise, ∆pH—change in pH in response to exercise; ** *p* < 0.01, *** *p* < 0.001—significant differences between normoxia and hypoxia; ^#^ *p* < 0.05, ^###^ *p* < 0.001—significant differences between groups (UT vs. T).

**Table 2:** Echocardiography-derived LV dimensions and systolic function parameters before and after exercise under normoxia and hypoxia in the untrained (UT, n = 12) and trained (T, n = 12) group.

Variable	Group	Normoxia		Hypoxia (3000 m)		ANOVA Results	
		At Rest (*x* ± SD)	Post Ex. (*x* ± SD)	At Rest (*x* ± SD)	Post Ex. (*x* ± SD)	Exercise	Exercise & Conditions
IVS (mm)	UT	10.08 ±0.746	10.55 ±0.80 **	10.25 ±0.95	10.45 ±0.94	*F* = 25.656 *p* = 0.001	*F* = 4.105 *p* = 0.050
	T	10.90 ±1.16	10.92 ±1.08	10.94 ±0.96	11.41 ±0.95	*F* = 2.100 *p* = 0.161	*F* = 1.820 *p* = 0.190
PW (mm)	UT	10.17 ±0.79	10.30 ±0.78	10.22 ±0.97	9.99 ±0.99	*F* = 0.123 *p* = 0.729	*F* = 1.877 *p* = 0.184
	T	11.08 ±1.06	11.18 ±0.76	10.73 ±0.72	11.13 ±0.99	*F* = 2.326 *p* = 0.141	*F* = 0.839 *p* = 0.369
LVEDD (mm)	UT	53.75 ±2.96	53.00 ±3.38	54.16 ±3.27	52.41 ±3.20 **	*F* = 11.702 *p* = 0.002	*F* = 1.872 *p* = 0.185
	T	53.15 ±3.28	51.47 ±2.78	49.44 ±14.38	51.47 ±3.66	*F* = 0.009 *p* = 0.925	*F* = 0.978 *p* = 0.333
LVESD (mm)	UT	35.67 ±2.50	35.00 ±3.52	35.33 ±2.42	35.17 ±3.43	*F* = 0.514 *p* = 0.480	*F* = 0.185 *p* = 0.671
	T	32.50 ± 3.29	34.64 ±3.03	32.73 ±2.83	34.07 ±3.13	*F* = 8.972 *p* = 0.007	*F* = 0.485 *p* = 0.493
LVEDV (mL)	UT	154.08 ±29.38	148.52 ±41.91	161.25 ±30.38	146.25 ±31.42 ***	*F* = 14.328 *p* = 0.001	*F* = 3.022 *p* = 0.096
	T	162.33 ±29.92	146.58 ±22.920	160.17 ±25.09	154.41 ±30.15	*F* = 7.183 *p* = 0.013	*F* = 1.553 *p* = 0.225
LVESV (mL)	UT	51.97 ±10.38	54.18 ±19.35	51.08 ±14.78	52.29 ±18.19	*F* = 0.512 *p* = 0.481	*F* = 0.043 *p* = 0.835
	T	52.66 ±11.48	55.09 ±9.68	51.07 ±10.78	56.06 ±14.48	*F* = 3.870 *p* = 0.061	*F* = 0.461 *p* = 0.504
LV EF (%)	UT	65.71 ±5.58	64.00 ±5.57	68.46 ±5.16	65.92 ±6.99	*F* = 3.392 *p* = 0.790	*F* = 0.128 *p* = 0.723
	T	67.1 ± 4.45	62.3 ± 3.93 *	68.21 ±4.83	64.1 3±5.14	*F* = 16.190 *p* = 0.001	*F* = 0.108 *p* = 0.745
LV GLS (%)	UT	−19.95 ±2.44	−14.92 ±2.57 ***	−20.30 ±2.45	−15.25 ±2.17 **	*F* = 88.433 *p* = 0.001	*F* = 0.001 *p* = 0.981
	T	−22.41 ±3.24	−15.55 ±2.87 ***	−22.37 ±2.34	−17.82 ±2.15 ***	*F* = 149.664 *p* = 0.001	*F* = 6.163 *p* = 0.021
Septal S′ (cm/s)	UT	8.85 ±1.12	8.61 ±2.25	8.96 ±0.72	8.72 ±1.37	*F* = 0.526 *p* = 0.475	*F* = 0.001 *p* = 0.995
	T	9.30 ±1.06	8.16 ±1.01	9.66 ±1.03	8.46 ±1.13 ***	*F* = 26.057 *p* = 0.001	*F* = 0.019 *p* = 0.890
Lateral S′ (cm/s)	UT	11.74 ±2.37	11.52 ±1.89	11.94 ±2.37	12.41 ±2.27	*F* = 0.065 *p* = 0.800	*F* = 0.480 *p* = 0.495
	T	12.66 ±3.43	10.64 ±2.50 *	12.10 ±3.07	11.49 ±2.88	*F* = 8.354 *p* = 0.001	*F* = 2.404 *p* = 0.135

Abbreviations: IVS—interventricular septum, PW—left ventricular posterior wall, LVEDD—left ventricular end-diastolic diameter, LVESD—left ventricular end-systolic diameter, LVEDV—left ventricular end-diastolic volumes, LVESV—left ventricular end-systolic volumes, LV EF—left ventricular ejection fraction, LV GLS—left ventricular global longitudinal strain, Septal S′—septal peak systolic mitral annulus velocity, Lateral S′—lateral peak systolic mitral annulus velocity; * *p_Bonf* < 0.05, ** *p_Bonf* < 0.01, *** *p_Bonf* < 0.001—significant differences after exercise (at rest vs. post exercise).

**Table 3:** Echocardiographic parameters of LV diastolic function and LA dimensions in the untrained (UT, n = 12) and trained (T, n = 12) group.

Variable	Group	Normoxia		Hypoxia (3000 m)		ANOVA Results	
		At Rest (x ± SD)	Post Ex. (x ± SD)	At Rest (x ± SD)	Post Ex. (x ± SD)	Exercise	Exercise & Conditions
Mitral E (cm/s)	UT	84.49 ±18.66	66.57 ±18.58 *	86.21 ±15.25	72.21 ±15.49 *	*F* = 27.093 *p* = 0.001	*F* = 0.408 *p* = 0.529
	T	84.77 ±15.39	65.05 ±13.19 **	87.15 ±13.20	74.19 ±16.13 **	*F* = 38.693 *p* = 0.001	*F* = 1.655 *p* = 0.211
Mitral A (cm/s)	UT	51.99 ±12.95	47.01 ±9.07	53.65 ±9.71	50.36 ±13.17	*F* = 1.305 *p* = 0.266	*F* = 0.178 *p* = 0.677
	T	49.15 ±17.85	51.08 ±12.77	51.98 ±10.98	53.91 ±14.29	*F* = 0.330 *p* = 0.571	*F* = 0.000 *p* = 0.999
E/A	UT	1.71 ±0.62	1.38 ±0.33	1.65 ±0.41	1.44 ±0.37	*F* = 7.305 *p* = 0.013	*F* = 0.606 *p* = 0.445
	T	1.74 ±0.43	1.31 ±0.29 *	1.76 ±0.50	1.41 ±0.30	*F* = 12.811 *p* = 0.001	*F* = 0.163 *p* = 0.690
DT (ms)	UT	195.82 ±53.02	195.27 ±52.31	208.27 ±39.06	183.73 ±38.76	*F* = 0.977 *p* = 0.334	*F* = 0.894 *p* = 0.355
	T	213.91 ±51.57	177.75 ±40.49	229.08 ±33.06	193.75 ±57.80	*F* = 7.225 *p* = 0.013	*F* = 0.001 *p* = 0.975
E′ septal (cm/s)	UT	12.66 ±2.40	10.82 ±2.219	12.64 ±1.33	10.84 ±1.99	*F* = 12.464 *p* = 0.001	*F* = 0.002 *p* = 0.967
	T	13.19 ±1.96	10.42 ± 2.32 **	12.23 ±2.05	10.53 ±1.61 *	*F* = 30.743 *p* = 0.001	*F* = 1.758 *p* = 0.198
E′ lateral (cm/s)	UT	19.11 ±5.14	15.42 ±3.26	18.34 ±4.96	14.79 ±3.97	*F* = 14.207 *p* = 0.001	*F* = 0.004 *p* = 0.946
	T	18.25 ±3.96	14.76 ±3.09 **	18.24 ±2.90	16.08 ±3.51	*F* = 22.693 *p* = 0.001	*F* = 1.263 *p* = 0.273
Averaged E′ (cm/s)	UT	15.88 ±3.29	13.12 ±2.46 *	15.49 ±2.87	12.82 ±2.71	*F* = 19.752 *p* = 0.001	*F* = 0.005 *p* = 0.964
	T	15.72 ±2.50	12.59 ±2.29 **	15.21 ±2.19	13.31 ±2.23 *	*F* = 33.618 *p* = 0.001	*F* = 2.011 *p* = 0.170
A′ septal (cm/s)	UT	8.49 ±1.52	7.89 ±1.45	8.71 ±1.86	8.42 ±1.66	*F* = 1.672 *p* = 0.209	*F* = 0.216 *p* = 0.645
	T	7.21 ±2.78	7.02 ±2.58	7.92 ±1.24	7.25 ±1.61	*F* = 0.616 *p* = 0.440	*F* = 0.190 *p* = 0.667
A′ lateral (cm/s)	UT	7.71 ±1.24	7.66 ±2.45	7.12 ±1.82	6.49 ±1.87	*F* = 0.340 *p* = 0.565	*F* = 0.244 *p* = 0.626
	T	7.86 ±2.11	7.18 ±3.20	7.51 ±1.83	6.75 ±3.26	*F* = 1.158 *p* = 0.293	*F* = 0.003 *p* = 0.951
Averaged A′ (cm/s)	UT	8.10 ±1.14	7.91 ±1.54	7.91 ±1.49	7.45 ±1.51	*F* = 1.016 *p* = 0.324	*F* = 0.015 *p* = 0.903
	T	7.54 ±2.02	7.10 ±2.80	7.72 ±1.47	7.01 ±1.99	*F* = 1.148 *p* = 0.295	*F* = 0.068 *p* = 0.795
Averaged E′/A′	UT	1.97 ±0.36	1.73 ±0.57	2.04 ±0.58	1.75 ±0.40	*F* = 4.063 *p* = 0.056	*F* = 0.145 *p* = 0.707
	T	2.46 ±1.36	2.10 ±1.20	2.01 ±0.61	2.04 ±0.69	*F* = 0.355 *p* = 0.556	*F* = 0.534 *p* = 0.472
E/averaged E′	UT	5.37 ±0.97	5.10 ±1.12	5.53 ±1.55	5.77 ±2.07	*F* = 0.001 *p* = 0.966	*F* = 0.483 *p* = 0.494
	T	5.50 ±1.20	5.26 ±1.21	5.37 ±1.81	5.60 ±1.09	*F* = 0.001 *p* = 0.989	*F* = 0.593 *p* = 0.449
LA diameter (mm)	UT	39.83 ±2.88	39.04 ±3.90	40.33 ±4.11	38.37 ±4.47 *	*F* = 9.377 *p* = 0.005	*F* = 1.688 *p* = 0.207
	T	39.25 ±2.87	36.86 ±3.15 *	38.43 ±2.93	38.28 ±3.12	*F* = 8.689 *p* = 0.007	*F* = 6.759 *p* = 0.016
LA area (cm^2^)	UT	25.21 ±4.08	22.21 ±4.57 *	24.17 ±3.81	22.53 ±4.49	*F* = 17.453 *p* = 0.001	*F* = 1.505 *p* = 0.232
	T	23.52 ±1.93	21.82 ±2.08	23.45 ±1.86	21.56 ±3.72	*F* = 11.571 *p* = 0.002	*F* = 0.027 *p* = 0.869
LAVi (mL/m^2^)	UT	40.77 ±9.52	32.06 ±9.49 **	41.90 ±12.05	34.07 ±9.25 *	*F* = 30.883 *p* = 0.001	*F* = 1.307 *p* = 0.265
	T	38.97 ±3.75	33.14 ±5.04 ***	38.97 ±4.77	33.87 ±7.67	*F* = 27.215 *p* = 0.001	*F* = 0.122 *p* = 0.729

Abbreviations: Mitral E—early peak diastolic filling velocity, Mitral A—late peak diastolic filling velocity, DT—deceleration time, E′ septal—septal early diastolic mitral annulus velocity, E′ lateral—lateral early diastolic mitral annulus velocity, Averaged E′—mean early diastolic mitral annulus velocity, A′ septal—septal late diastolic mitral annulus velocity, A′ lateral—lateral late diastolic mitral annulus velocity, Averaged A′—mean late diastolic mitral annulus velocity, Averaged E′/A′—the ratio of early to late annular velocity, E/averaged E′—the ratio of transmitral E velocity to early diastolic mitral annular velocity, LA diameter—left atrial diameter, LA area—left atrial area, LAVi—left atrial indexed volume; * *p_Bonf* < 0.05, ** *p_Bonf* < 0.01, *** *p_Bonf* < 0.001—significant differences after exercise (at rest vs. post exercise).

**Table 4:** Comparison of delta (∆) values of selected variables in response to exercise to exhaustion between untrained (UT, n = 12) and trained (T, n = 12) men under different conditions (normoxia vs. hypoxia). The table presents only ∆ values for which significant differences were observed.

Variable	Group	Normoxia	Hypoxia (3000 m)
∆LA diameter (mm)	UT	−0.79 ± 2.19	−1.95 ± 2.19
	T	−2.39 ± 2.53	−0.15 ± 1.57 ^#^
∆PW (mm)	UT	0.13 ± 0.55	−0.22 ± 0.71
	T	0.10 ± 0.95	0.40 ± 0.62 ^#^
∆LVESD (mm)	UT	−0.66 ± 2.77	−0.16±2.91
	T	2.14 ± 2.76 ^#^	1.33 ± 2.91
∆LV EF (%)	UT	−1.71 ± 3.79	−2.53 ± 7.02
	T	−4.80 ± 4.34 ^#^	−4.07 ± 6.28
∆Septal S′ (cm/s)	UT	−0.24 ± 1.96	−0.23 ± 1.20
	T	−1.14 ± 1.41	−1.20 ± 0.73 ^#^

Abbreviations: Change in left atrial diameter (∆LA diameter), left ventricular posterior wall (∆PW), left ventricular end-systolic diameter (∆LVESD), left ventricular ejection fraction (∆LV EF) and septal peak systolic mitral annulus velocity (∆Septal S′) in response to exercise; ^#^ *p* < 0.05—significant differences between groups (UT vs. T).

## Data Availability

The data presented in this study are available on request from the corresponding author.

## Abbreviations

The following abbreviations are used in this manuscript:

EICF	Exercise-induced cardiac fatigue
LV	Left ventricular
IVS	Interventricular septum
PW	Left ventricular posterior wall
LVEDD	Left ventricular end-diastolic diameter
LVESD	Left ventricular end-systolic diameter
LVEDV	Left ventricular end-diastolic volumes
LVESV	Left ventricular end-systolic volumes
LV EF	Left ventricular ejection fraction
LV GLS	Left ventricular global longitudinal strain
Septal S′	Septal peak systolic mitral annulus velocity
Lateral S′	Lateral peak systolic mitral annulus velocity
Mitral E	Early peak diastolic filling velocity
Mitral A	Late peak diastolic filling velocity
DT	Deceleration time
E′ septal	Septal early diastolic mitral annulus velocity
E′ lateral	Lateral early diastolic mitral annulus velocity
Averaged E′	Mean early diastolic mitral annulus velocity
A′ septal	Septal late diastolic mitral annulus velocity
A′ lateral	Lateral late diastolic mitral annulus velocity
Averaged A′	Mean late diastolic mitral annulus velocity
LA diameter	Left atrial diameter
LA area	Left atrial area
LAVi	Left atrial indexed volume
GXT	Graded exercise test to volitional exhaustion
CXT	Constant-workload exercise test, continued until volitional exhaustion
ICC	Intraclass correlation coefficients
CI	Confidence interval
